# Changes and Compensatory Gait Mechanisms in Lower Extremity Kinematics in Patients With ACL Rupture: A Cross-sectional Controlled Study

**DOI:** 10.1177/19417381261479683

**Published:** 2026-09-11

**Authors:** Xuan Zhao, Danni Wu, Shaobai Wang, Peter Theobald

**Affiliations:** † School of Engineering, Cardiff University, Cardiff, UK; ‡ School of Exercise and Health, Shanghai University of Sport, Shanghai, China

**Keywords:** ACL rupture, gait analysis, lower extremity kinematics, rehabilitation, wearable sensors

## Abstract

**Background:**

Anterior cruciate ligament (ACL) rupture is associated with knee instability and compensatory gait adaptations, which are relevant to rehabilitation planning.

**Hypothesis:**

Patients with ACL rupture would show altered bilateral hip, knee, and ankle gait kinematics.

**Study Design:**

Cross-sectional study.

**Level of Evidence:**

Level 3.

**Methods:**

A total of 40 participants were enrolled, including 22 patients with recent ACL ruptures and 18 healthy subjects. Participants walked on a treadmill at 4 km/h while wearing 17 inertial sensors. Ten normalized gait cycles were used to compare bilateral hip, knee, and ankle kinematics.

**Results:**

Patients with ACL rupture demonstrated altered lower-extremity kinematics compared with healthy subjects. Hip external rotation was reduced bilaterally (*P* < 0.01), and hip adduction was reduced on both the affected (*P* < 0.05) and nonaffected (*P* < 0.01) sides. Maximum knee flexion was lower on the affected side than in healthy subjects (*P* < 0.01) and the nonaffected side (*P* < 0.05). Maximum ankle internal rotation was reduced bilaterally (affected side *P* < 0.05; nonaffected side *P* < 0.01), suggesting bilateral gait adaptations after ACL rupture.

**Conclusion:**

ACL rupture was associated with gait alterations involving both lower extremities, with bilateral hip and ankle kinematic changes and reduced maximum knee flexion mainly on the affected side.

**Clinical Relevance:**

Recognition of bilateral kinematic changes after ACL rupture may help clinicians assess both limbs and guide rehabilitation focused on restoring functional gait patterns.

More than 2 million anterior cruciate ligament (ACL) injuries occur each year globally,^16^ with a mean ACL rupture rate of 1 in 3500.^5^ The most common causes of ACL rupture during athletic activity include sudden deceleration, cutting stresses, and excessive knee rotation. Due to the integrative nature of the lower extremity kinetic chain, abnormal activity in the hips and ankles may also increase ACL injury risk.^13^ Because the ACL contributes to knee stability, ACL rupture is commonly associated with knee instability and may be accompanied by consequential injuries, including osteoarthritis and meniscal tears.^11,15^

Gait phasing allows joint-angle changes to be examined in predefined portions of the gait cycle, helping to identify phase-specific movement strategies.^1,10,19^ ACL rupture patients often have reduced peak knee flexion during walking, along with changes in internal and external rotation angles.^9,17^ These knee kinematic changes may be accompanied by adaptations in adjacent joints, including the hip and ankle.^12^ In this study, potential compensatory gait adaptations were operationally considered as hip, knee, or ankle kinematic deviations in the ACL-ruptured limb or the contralateral nonaffected limb compared with healthy subjects. Related knee-rehabilitation research has also emphasized the clinical relevance of lower-extremity alignment correction,^14^ although its relationship with acute ACL-rupture gait adaptations remains unclear. Therefore, it remains important to clarify how bilateral hip, knee, and ankle kinematics change after ACL rupture across predefined gait phases.

Wearable inertial measurement unit (IMU) based motion capture is commonly used to quantify gait kinematics and has shown good agreement with optical motion capture systems^4^; the coefficient of multiple correlation is 0.87 to 0.97.^8,21^ By mapping sensor data onto body segments, IMU systems allow lower-extremity joint kinematics to be estimated during walking.

This study aims to use wearable IMU based sensors to compare bilateral hip, knee, and ankle gait kinematics among the ACL-ruptured limb, the contralateral nonaffected limb, and healthy subjects. These findings may provide context for clinical assessment and rehabilitation planning after ACL rupture.

## Methods

### Ethics Statement

Ethical approval for this study was obtained from the Ethics Committee of Shanghai University of Sport (approval number: 102772023RT039). The study was performed in accordance with the Declaration of Helsinki, and in accordance with the local legislation and institutional requirements. The participants provided their written informed consent to participate in this study.

### Participants

A total of 40 participants were recruited, comprising 18 healthy subjects and 22 patients. Demographic data are summarized in Table 1, with the mean age of all subjects being 24.3 ± 3.8 years, mean height 171.4 ± 8.1 cm, mean weight 65.5 ± 11.6 kg, and mean body mass index (BMI) 22.1 ± 2.4 kg/m^2^.

**Table 1.** Demographics of participants

Group	Sex, male:female	Age, years	Height, cm	Weight, kg	BMI, kg/m^2^
Healthy subjects (n = 18)	12:6	24.7 ± 2.3	170.6 ± 8.1	63.7 ± 10.3	21.7 ± 1.8
Patients (n = 22)	14:8	24.0 ± 4.8	172.1 ± 8.3	67.0 ± 12.6	22.5 ± 2.8
All (n = 40)	26:14	24.3 ± 3.8	171.4 ± 8.1	65.5 ± 11.6	22.1 ± 2.4
*P* value	0.84	0.59	0.56	0.38	0.36

Data given as (mean ± SD). BMI, body mass index.

Participants in the patient group were patients with complete ACL rupture. All were recruited within 3 days before ACL reconstruction surgery, and physical examination confirmed positive Lachman tests indicative of ACL deficiency. Inclusion required compliance with all criteria: (1) age between 18 and 35 years; (2) confirmed diagnosis via magnetic resonance imaging scan of complete ACL rupture; (3) injury resulting from acute, closed trauma during sports activity; (4) absence of concomitant knee injuries; and (5) no other sports trauma within the past 6 months.

Healthy participants were university students. After a physical examination conducted by an orthopaedic physician, participants with no history of knee pathology or lower limb dysfunction were included. Inclusion criteria for healthy subjects comprised: (1) age between 18 and 35 years, (2) no history of bilateral knee injuries, and (3) no orthopaedic trauma or injury within the past 6 months.

Sample size estimation was conducted utilizing G*Power software (Version 3.1.9.2). An *F*-test for analysis of variance (ANOVA) was employed to calculate the required sample size based on preliminary data, which yielded an effect size of 0.65. Setting the significance level (α) at 0.05 and the statistical power (1 – β) at 0.80, the initial calculation indicated a total sample size of 21 participants. To account for potential variances and data collection issues, this study ultimately enrolled 22 patients.

### Gait Kinematics Collection

#### Sensor Configuration and Calibration

**Figure 1.**
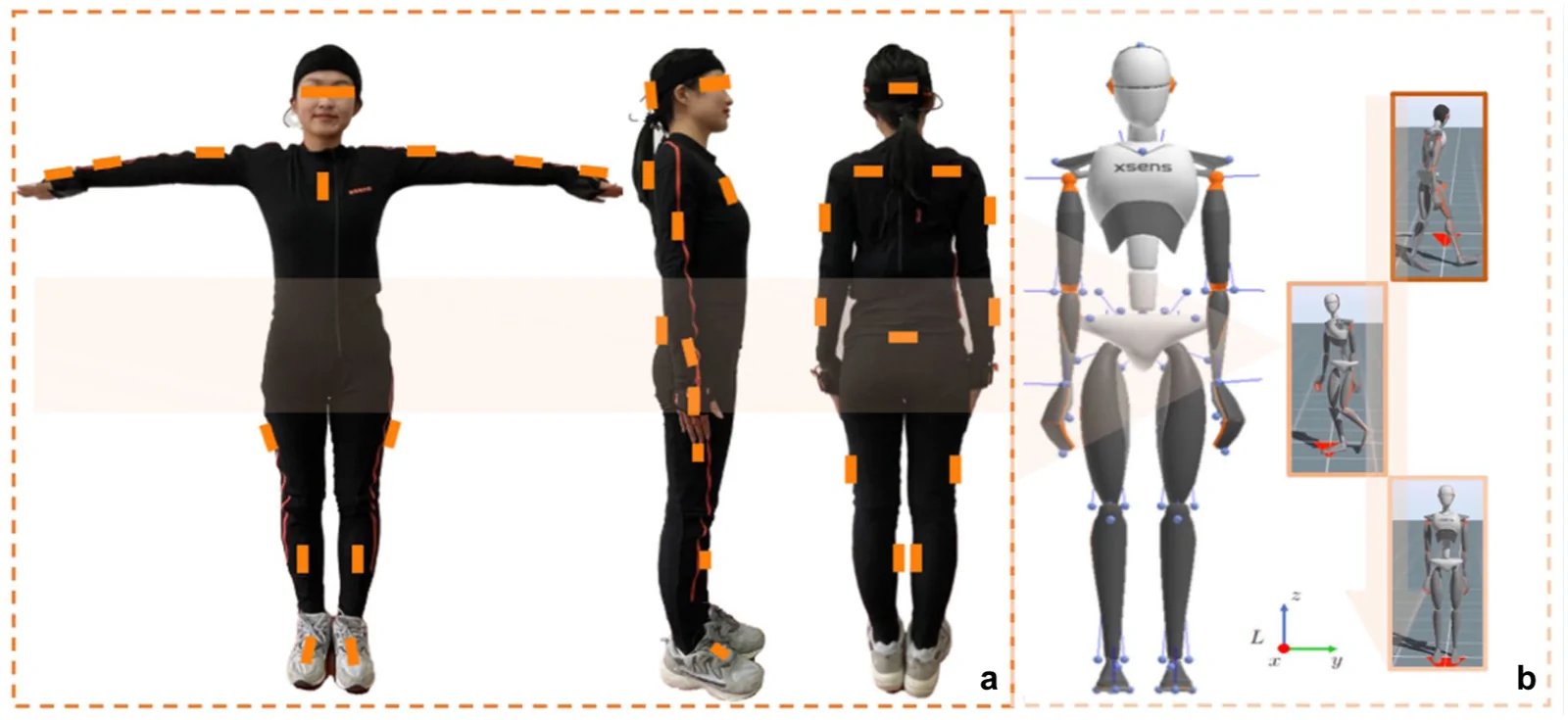
Sensor fixation and model construction. (a) Placement of IMUs of the MVN Link System on the body. (b) Process of model construction, including the establishment of joint coordinates essential for accurate kinematic analysis. IMU, inertial measurement unit.

Participants donned a specialized Lycra suit designed to affix a total of 17 IMUs at specified anatomical sites (Figure 1a) (MVN LINK, Xsens Technologies BV). Each IMU was connected via cable to a central data acquisition unit, with the main console and power supply fixed at the back of the waist. Cables were secured tidily in the Lycra suit to prevent movement artifacts. Sensor data are transmitted wirelessly through a router to a computer system. Each IMU includes a gyroscope, an accelerometer, and a magnetometer. Participants’ lower limb and foot lengths were recorded to facilitate model scaling.

**Figure 2.**
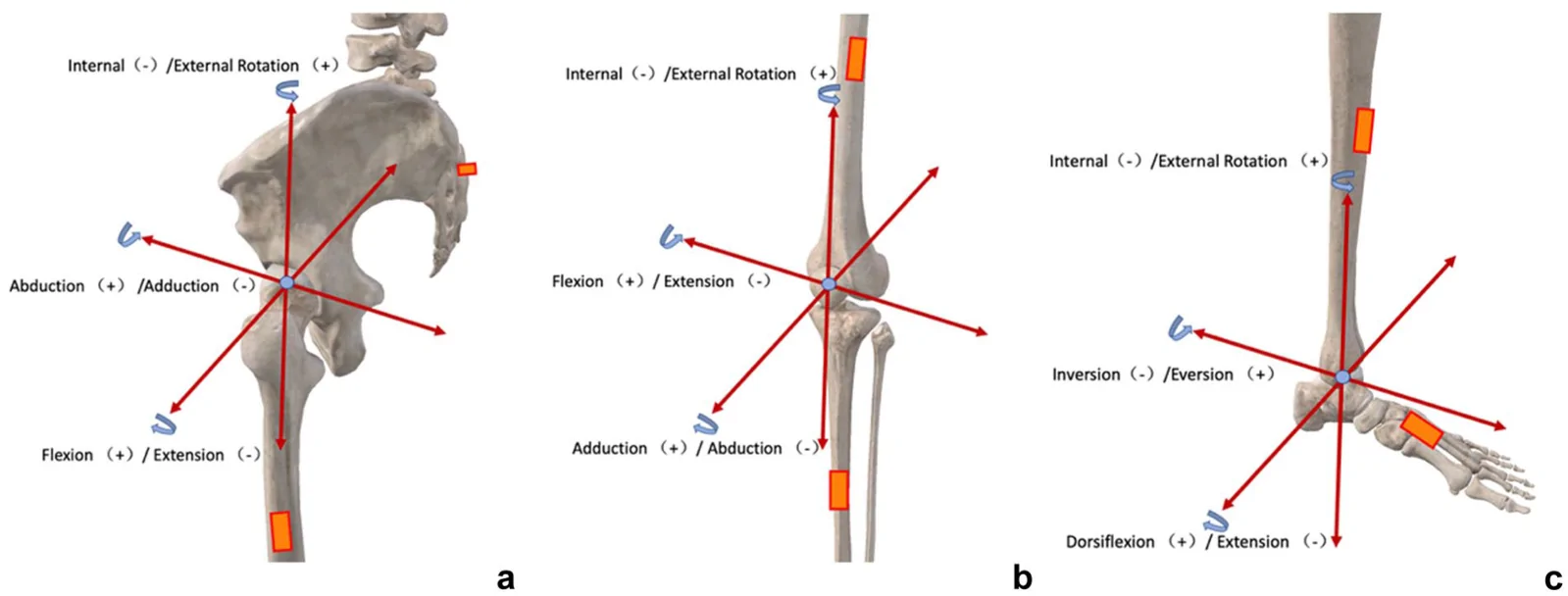
Schematic of lower extremity joint sensors and movement coordinate systems. Orange squares represent sensors. (a) Pelvic skeletal model with sensor placement at the bilateral posterior superior iliac spines and the lateral midsegment of the femur. (b) Knee model with sensors positioned on the lateral midsegment of the femur and the anterior midsegment of the tibia. (c) Ankle model with sensors placed on the anterior midsegment of the tibia and the dorsal surface of the foot.

During calibration, the relative positions between adjacent sensors were recorded, and their spatial changes over time were used to compute the 3 degrees of freedom (DOF) joint kinematics. Sensor placement and DOFs measured at lower extremity joints were as follows: (1) hips: positioned on the pelvis at the posterior superior iliac spine line and on the lateral midsegment of the femur (bilaterally, 1 each) (Figure 2a). (2) knees: positioned on the lateral midsegment of the femur (bilaterally, 1 each) and the anterior midsegment of the tibia (bilaterally, 1 each) (Figure 2b). (3) ankles: positioned on the anterior midsegment of the tibia (bilaterally, 1 each) and the dorsal aspect of the foot (bilaterally, 1 each) (Figure 2c). All joints included 3 DOF in movement.

#### Instrument Validity and Operator Reliability

Previous validation studies have reported acceptable-to-excellent agreement between IMU-based systems and optical motion capture systems for lower-extremity kinematic assessment,^3,21^ primarily in healthy adults during gait and functional movement tasks. Although IMU based gait analysis has been used in patients with ACL rupture,^20^ no separate concurrent validation of the present protocol against optical motion capture was performed in patients with acute complete ACL rupture.

To minimize operator-related variability, all sensor placement, calibration, gait-cycle selection, and data processing were performed by the same trained operator according to a predefined protocol. Because no second operator or repeated testing session was included, study-specific inter- and intrarater reliability were not calculated.

#### Kinematic Data Collection

**Figure 3.**
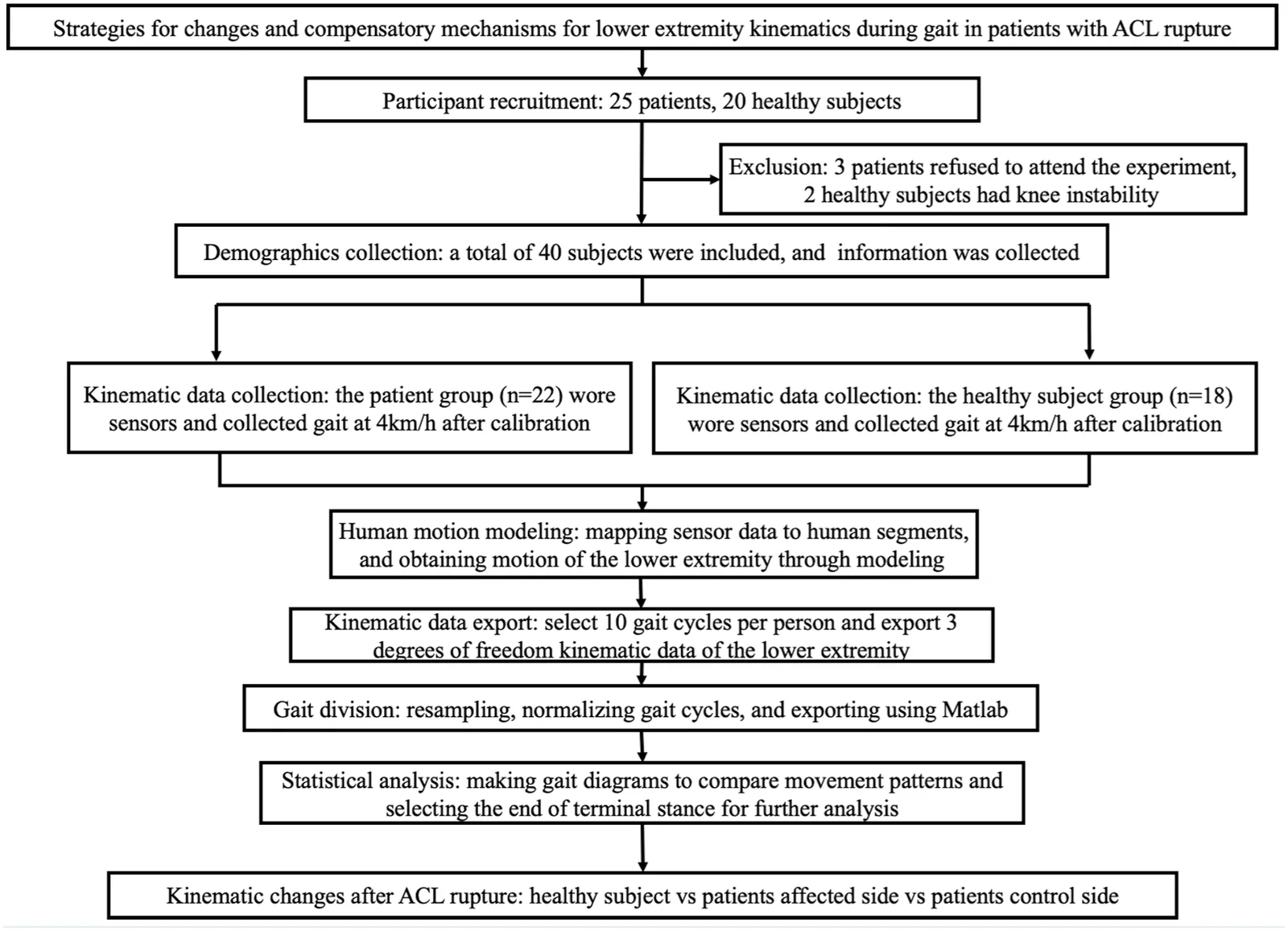
Study design and data analysis workflow. Flowchart summarizing the recruitment of patients with ACL rupture and healthy subjects, data collection procedures, and subsequent analysis steps. Participants were recruited, screened, and allocated to the patient (n = 22) or healthy subject (n = 18) group. Kinematic data were collected during gait, followed by human motion modeling, gait cycle segmentation, and normalization. Final analyses included statistical comparisons of gait kinematics between patients and healthy subjects, as well as between the affected and nonaffected sides in patients. ACL, anterior cruciate ligament.

The MVN LINK (Xsens Technologies BV) system was configured to operate at a sampling frequency of 240 Hz. The data collection procedure was standardized as follows (Figure 3). First, participants’ lower-limb and foot lengths were recorded for model scaling. Second, participants wore the Lycra suit, and 17 IMUs were fixed at predefined anatomical locations. Third, system calibration was performed, and participants completed static standing, straight-line walking, and turning movements to verify sensor stability and the absence of visible displacement (Figure 1b). Fourth, participants walked on the treadmill (Keep K3, Beijing Calorie Technology Co) with the speed gradually increased to 4 km/h. Fifth, after approximately 3 minutes of treadmill familiarization and confirmation of a stable gait pattern, kinematic data were recorded continuously for 2 minutes.

#### Data Processing

A biomechanical model of each participant was created by scaling the preloaded limb segments using the proprietary software (MVN Analyze Pro 2022, Xsens Technologies BV). Sensor data were mapped onto the corresponding segments of each model, which was established according to the standards of the International Society of Biomechanics. Subsequently, lower extremities kinematic data were derived from the model for analysis.

**Figure 4.**
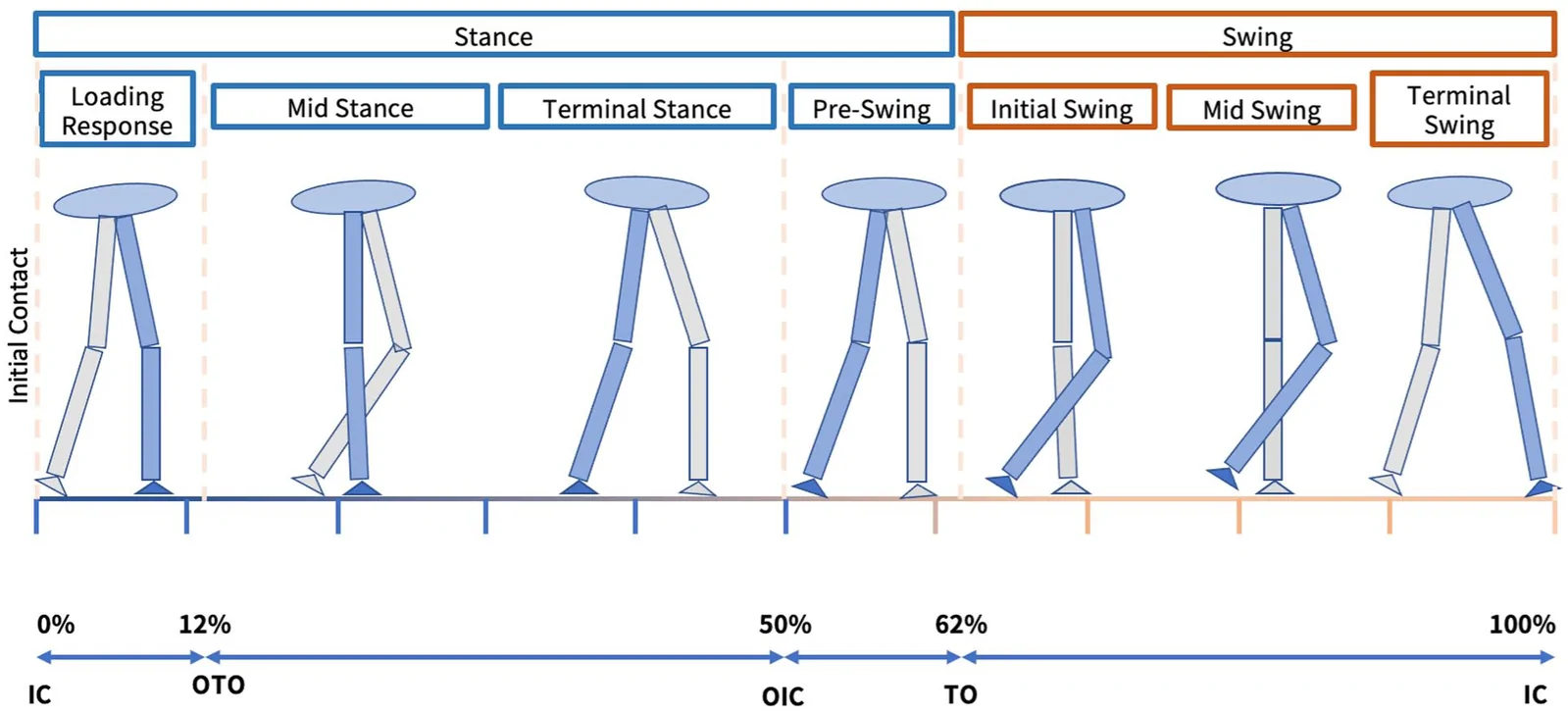
Division of the gait cycle and corresponding phases. Schematic representation of the gait cycle divided into stance (loading response, mid stance, terminal stance, and pre-swing) and swing (initial swing, mid swing, and terminal swing) phases. Percentages along the bottom indicate the relative timing of each phase within a complete gait cycle (0% to 100%). IC, initial contact; OIC, opposite initial contact; OTO, opposite toe-off; TO, toe-off.

Ten complete gait cycles were selected for analysis from each participant and the kinematic data exported describing hips, knees, and ankles. The joint kinematic data were resampled, and all gait cycles were normalized to 100 frames, representing 100% of the gait cycle, using MATLAB (R2016a, The MathWorks Inc). A gait cycle was defined as the interval from the heel strike of one foot to the subsequent heel strike of the same foot. Each cycle comprises phases,^1,19^ including stance (0% to 61%) and swing (62% to 100%) (Figure 4). The cycle begins at the affected heel contact (0%) and continues until the nonaffected toe-off (12%) as the loading response period. The mid- to terminal-stance is from nonaffected toe-off (12%) to this heel strike (50%). Pre-swing is from nonaffected heel strike (50%) to affected toe-off (62%), marking the end of stance. The moment of 50% gait cycle is when the terminal stance turns into pre-swing. The swing phase starts at affected toe-off (62%) and ends at the subsequent this heel strike (100%), which can be subdivided further into initial, mid, and terminal swing stages.

### Statistical Analysis

Data analysis was performed using SPSS Version 26.0 (IBM Corp). The Shapiro-Wilk test was used to assess the normality of data distributions across groups. Group comparisons at specific gait events were performed using 1-way ANOVA, followed by least significant difference (LSD) post hoc tests when the omnibus test was significant. As sensitivity analyses, Welch ANOVA and Kruskal-Wallis tests were performed for the main kinematic outcomes to assess the robustness of the omnibus group differences. Statistical significance was set at *P* < 0.05.

## Results

### Hip Kinematics

**Figure 5.**
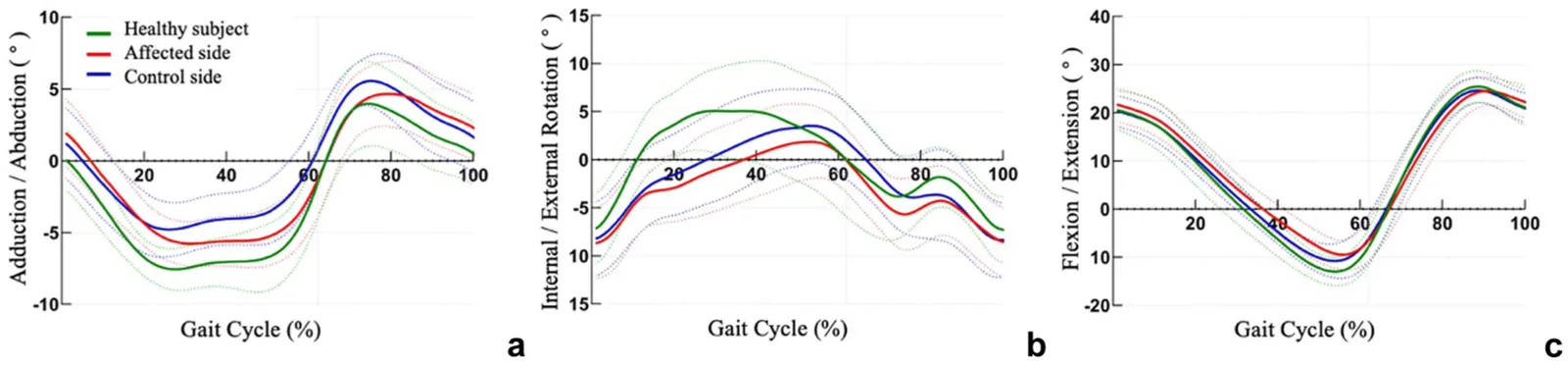
Gait diagram of hip kinematics. Mean hip joint angle changes throughout the gait cycle with standard deviations. Green lines, healthy subjects; red lines, affected side of patients with ACL rupture; blue lines, control side. (a) Adduction-abduction angles. (b) Internal-external rotation angles. (c) Flexion-extension angles. ACL, anterior cruciate ligament.

The hips exhibit 3 DOF kinematics during gait (Figure 5). Significant group differences were observed in maximum external rotation (*F* = 10.814; *P* < 0.001), adduction/abduction at 50% of the gait cycle (*F* = 9.554; *P* < 0.001), and flexion/extension at 50% of the gait cycle (*F* = 5.187; *P* = 0.008) (Tables 2 and 3). Post hoc comparisons showed reduced maximum external rotation bilaterally compared with healthy subjects (both *P* < 0.01). At 50% of the gait cycle, adduction and/or abduction differed between healthy subjects and both the affected (*P* < 0.05) and nonaffected (*P* < 0.01) sides, and also between the affected and nonaffected (*P* < 0.05) sides. Flexion and/or extension differed between the affected side and healthy subjects (*P* < 0.01). Other hip parameters did not differ significantly among groups.

**Table 2.** Comparison of key kinematic hip parameters

Group	N	Maximum external rotation at 26%, deg	Maximum abduction at 74%, deg	Maximum flexion at 86%, deg
Healthy	18	4.74 ± 3.84	3.93 ± 3.10	24.62 ± 3.56
Affected	22	–2.00 ± 4.51*	4.14 ± 2.57	22.69 ± 3.91
Nonaffected	22	–0.82 ± 5.69*	5.45 ± 1.83	23.80 ± 3.23
*F* value	-	10.814	2.232	1.466
*P* value	-	<0.001	0.12	0.24

Data given as (mean ± SD).

* Statistically significantly different compared with the healthy subject group (*P* < 0.01).

**Table 3.** Comparison of hip kinematics at 50% gait cycle

Group	N	Adduction/abduction, deg	Internal/external, deg	Flexion/extension, deg
Healthy	18	–6.83 ± 2.36	3.67 ± 5.86	–11.84 ± 2.99
Affected	22	–5.29 ± 2.11**	0.80 ± 4.90	–6.29 ± 5.42*
Nonaffected	22	–3.77 ± 2.19*, ***	3.08 ± 4.58	–8.52 ± 6.79
*F* value	-	9.554	1.838	5.187
*P* value	-	<0.001	0.17	0.008

Data given as (mean ± SD).

* Statistically significantly different compared with the healthy subject group (*P* < 0.01).

** Statistically significantly different compared with the healthy subject group (*P* < 0.05).

*** Statistically significantly different compared with the affected side group (*P* < 0.05).

### Knee Kinematics

**Figure 6.**
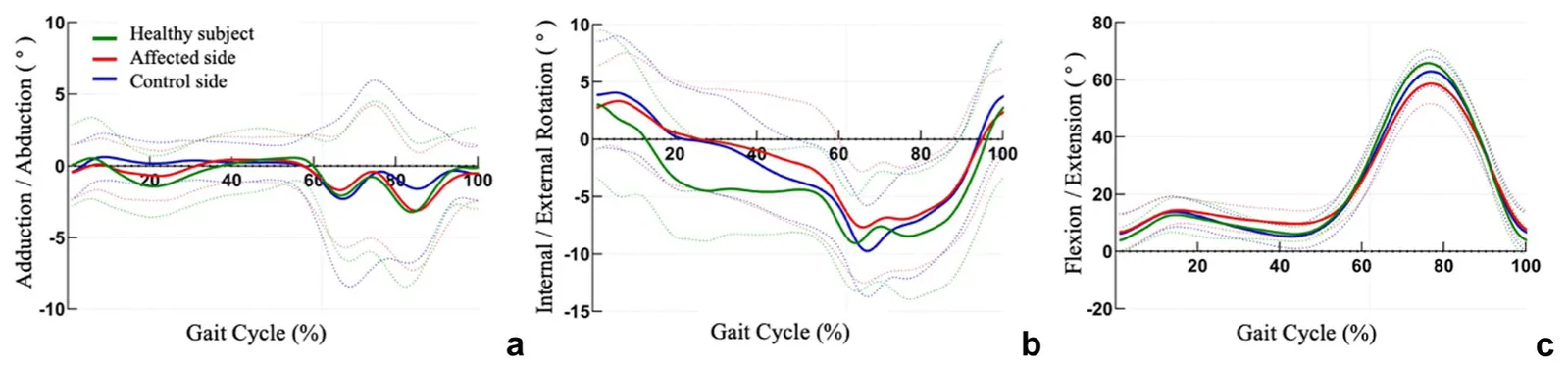
Gait diagram of the knee kinematics. Mean knee joint angle changes throughout the gait cycle with standard deviations. Green lines, healthy subjects; red lines, affected side of patients with ACL rupture; blue lines, control side. (a) Adduction-abduction angles. (b) Internal-external rotation angles. (c) Flexion-extension angles. ACL, anterior cruciate ligament.

The knees exhibit 3 DOF kinematics during gait (Figure 6). A significant group difference was observed only in maximum knee flexion at 76% of the gait cycle (*F* = 7.635; *P* = 0.001) (Table 4). Post hoc comparisons showed lower maximum flexion on the affected side than in healthy subjects (*P* < 0.01) and the nonaffected side (*P* < 0.05), whereas the nonaffected side did not differ from healthy subjects. At 50% of the gait cycle, no significant group differences were observed in knee adduction/abduction, internal/external rotation, or flexion/extension (all *P* > 0.05) (Table 5).

**Table 4.** Comparison of key kinematic knee parameters

Group	N	Maximum adduction at 84%, deg	Maximum internal rotation at 64%, deg	Maximum flexion at 76%, deg
Healthy	18	–2.95 ± 5.45	–8.49 ± 3.86	65.66 ± 4.76
Affected	22	–2.92 ± 4.52	–6.94 ± 5.83	58.06 ± 7.47*
Nonaffected	22	–1.44 ± 5.97	–7.91 ± 4.26	62.25 ± 5.40***
*F* value	-	0.548	0.531	7.635
*P* value	-	0.58	0.59	0.001

Data given as (mean ± SD).

* Statistically significantly different compared with the healthy subject group (*P* < 0.01).

*** Statistically significantly different compared with the affected side group (*P* < 0.05).

**Table 5.** Comparison of the knee kinematics at 50% gait cycle

Group	N	Adduction/abduction, deg	Internal/external rotation, deg	Flexion/extension, deg
Healthy	18	0.08 ± 2.95	–3.30 ± 4.41	7.18 ± 3.47
Affected	22	–0.04 ± 2.65	–0.88 ± 5.95	9.07 ± 5.82
Nonaffected	22	0.12 ± 1.37	–3.93 ± 4.86	7.34 ± 4.94
*F* value	-	0.026	2.105	0.941
*P* value	-	0.98	0.13	0.40

Data given as (mean ± SD).

### Ankle Kinematics

**Figure 7.**
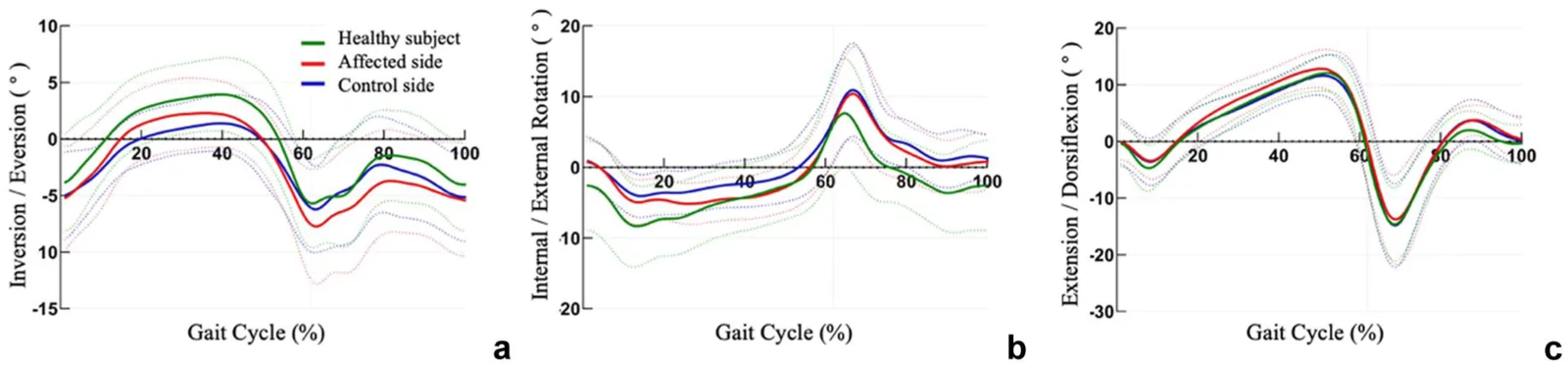
Gait diagram of the ankle kinematics. Mean ankle joint angle changes throughout the gait cycle with standard deviations. Green lines, healthy subjects; red lines, affected side of patients with ACL rupture; blue lines, control side. (a) Inversion-eversion angles. (b) Internal-external rotation angles. (c) Extension-dorsiflexion angles. ACL, anterior cruciate ligament.

The ankles exhibit 3 DOF kinematics during gait (Figure 7). Significant group differences were observed in maximum internal rotation at 13% of the gait cycle (*F* = 5.185; *P* = 0.008) (Table 6). Post hoc comparisons showed lower maximum internal rotation on both the affected (*P* < 0.05) and nonaffected (*P* < 0.01) sides compared with healthy subjects. A primary ANOVA difference was also observed for maximum eversion at 43% of the gait cycle (*F* = 3.259; *P* = 0.05), but this finding was not emphasized because it was not supported by sensitivity analyses (*P* = 0.06 and *P* = 0.08). At 50% of the gait cycle, no significant omnibus group differences were observed in ankle inversion/eversion, internal/external rotation, or dorsiflexion/extension (all *P* > 0.05) (Table 7).

**Table 6.** Comparison of key kinematic ankle parameters

Group	N	Maximum eversion at 43%, deg	Maximum internal rotation at 13%, deg	Maximum extension at 69%, deg
Healthy	18	3.88 ± 3.42	–8.18 ± 6.11	–14.53 ± 6.52
Affected	22	2.02 ± 3.33	–4.75 ± 3.77**	–13.55 ± 7.85
Nonaffected	22	1.33 ± 2.91**	–3.75 ± 3.48*	–14.67 ± 8.11
*F* value	-	3.259	5.185	0.139
*P* value	-	0.05	0.008	0.87

Data given as (mean ± SD).

* Statistically significantly different compared with the healthy subject group (*P* < 0.01).

** Statistically significantly different compared with the healthy subject group (*P* < 0.05).

**Table 7.** Comparison of ankle kinematics at 50% gait cycle

Group	N	Inversion/eversion, deg	Internal/external rotation, deg	Dorsiflexion/extension, deg
Healthy	18	2.58 ± 3.67	–2.76 ± 4.92	11.72 ± 2.95
Affected	22	0.37 ± 3.23	–3.06 ± 4.39	12.74 ± 3.34
Nonaffected	22	0.17 ± 3.64	–1.34 ± 3.83	11.45 ± 3.83
*F* value	-	2.782	0.965	0.866
*P* value	-	0.07	0.39	0.43

Data given as (mean ± SD).

### Sensitivity Analyses

Sensitivity analyses showed that the main omnibus findings for hip maximum external rotation (both *P* < 0.001), hip adduction/abduction at 50% of the gait cycle (both *P* < 0.001), hip flexion/extension at 50% of the gait cycle (both *P* < 0.001), knee maximum flexion (both *P* = 0.002), and ankle maximum internal rotation (*P* = 0.04 and *P* = 0.03) remained significant using Welch ANOVA and Kruskal-Wallis tests. The primary ANOVA finding for maximum ankle eversion was not significant in sensitivity analyses and was therefore not interpreted as a main finding (*P* = 0.06 and *P* = 0.08).

## Discussion

In this cross-sectional comparison, patients with ACL rupture showed joint- and phase-specific lower-extremity kinematic alterations. The main findings were: (1) bilateral reductions in hip external rotation and hip adduction, with reduced hip flexion on the affected side at 50% of the gait cycle; (2) reduced maximum knee flexion on the affected side compared with healthy subjects and the nonaffected side, whereas knee rotation and knee kinematics at 50% of the gait cycle did not show significant group differences; and (3) bilateral reductions in maximum ankle internal rotation. Overall, these findings suggest that gait adaptations after ACL rupture may involve both lower extremities, but the alterations were joint- and phase-specific rather than uniform across all kinematic parameters.

### Hip Kinematics

Hip kinematic alterations were observed mainly in external rotation and in adduction/abduction and flexion/extension at 50% of the gait cycle. The angle-time curves suggested delayed external rotation of the hip during the stance phase, particularly in the mid- to terminal-phase, and the peak external rotation angle was lower bilaterally than in healthy subjects. This finding is consistent with previous research reporting lower average hip rotation in patients with ACL rupture than in healthy subjects.^2^ The lower hip rotation might be associated with a late-onset internal-external rotation transition, which occurs during the stance phase, while maintaining the same phase-specific transition from external to internal rotation. A possible interpretation is that patients in the gait cycle may not reach their maximum external hip rotation, possibly associated with the need for internal rotation to maintain overall gait harmony and phase coordination. Clinically, this finding may inform preoperative gait assessment, but its implications for orthotic design require further investigation.

At 50% of the gait cycle, bilateral hip adduction angles differed between patients with ACL rupture and healthy subjects, with a greater difference on the nonaffected side than on the affected side. This contrasts with previous research, which reported that healthy people have smaller hip abduction/adduction mean angles compared with ACL injured subjects (*P* = 0.005),^2^ likely relevant to differences in analysis timing and methodology. Previous studies analyzed average hip activity over the entire gait cycle without stage-specific subdivisions, whereas our study focused specifically on the terminal stance phase. The affected side also showed altered hip flexion/extension at 50% of the gait cycle compared with healthy subjects. This phase-specific difference may reflect altered proximal limb positioning during terminal stance to pre-swing, but potential links with muscle strength or activation remain speculative because these variables were not measured.

Not all hip variables differed among groups. Maximum hip abduction and flexion, and hip internal/external rotation at 50% of the gait cycle were not significantly different. Therefore, the hip findings should be interpreted as parameter- and phase-specific rather than as a generalized restriction of hip motion. Clinically, these results may support considering hip kinematics during preoperative assessment and rehabilitation planning, but interventional studies are needed to determine whether hip-focused strategies can modify these gait patterns.

### Knee Kinematics

The main knee finding was reduced maximum knee flexion on the affected side during the swing phase. At 76% of the gait cycle, the affected side showed lower maximum flexion than both healthy subjects and the nonaffected side, whereas the nonaffected side did not differ significantly from healthy subjects. This finding is consistent with previous reports of reduced peak knee flexion after ACL injury,^23^ and may reflect protective gait behavior or acute-phase limitations in joint mobility.

The knee internal rotation angle-time curves showed a descriptive tendency toward delayed onset and reduced magnitude of internal rotation during stance, particularly from mid- to terminal-stance. This pattern aligns with previous research suggesting that patients with ACL deficiency may adopt rotational adaptations to avoid tibiofemoral instability.^7^ Rather than confirming a bilateral reduction in knee internal rotation, the present findings suggest a possible alteration in hip-knee coordination. Previous studies analyzing the effects of ACL injury on bilateral muscle activation have reported that, relative to the contralateral side, the co-contraction of the hamstrings and quadriceps muscles is increased markedly postinjury, particularly with greater prominence in the lateral hamstrings.^9^ These altered muscular synergies may be relevant to hip-knee coordination after ACL injury, but the present study did not assess muscle activation directly. Therefore, this mechanism remains speculative.

At 50% of the gait cycle, knee adduction/abduction, internal/external rotation, and flexion/extension did not differ significantly among groups. These nonsignificant findings suggest that knee kinematic differences were not present uniformly during terminal stance or pre-swing. Overall, knee alterations were most evident in affected-side maximum flexion during swing rather than in all knee motion components across the gait cycle. This affected-side reduction in maximum knee flexion may be relevant for preoperative gait assessment and rehabilitation planning, although interventional studies are needed to determine whether targeted proprioceptive or strengthening strategies can modify this kinematic pattern. Recent evidence also suggests that neuromuscular responses to knee-extension interventions may be protocol- and population-dependent.^6^

### Ankle Kinematics

This study observed a bilateral reduction in maximum internal rotation compared with healthy subjects, consistent with previous findings on ankle rotation patterns in children with ACL rupture during gait.^18^ The bilateral reduction in maximum internal rotation may represent a compensatory gait pattern related to knee stability,^22^ although the present cross-sectional study cannot determine the underlying mechanism.

At 50% of the gait cycle, ankle inversion/eversion, internal/external rotation, and dorsiflexion/extension did not show significant group differences. Therefore, the ankle findings should be interpreted as specific to maximum internal rotation rather than as a generalized restriction across all ankle kinematic parameters. Clinically, these results suggest that ankle kinematics may be considered during gait assessment after ACL rupture, while specific ankle-focused interventions require further study.

This study possesses several limitations. Due to the constraints of the experimental timeframe, data collection was conducted at a single preoperative time point, which limits the ability to observe longitudinal changes across different stages of injury and recovery. In addition, although a standardized protocol was used, the present IMU-based gait-analysis protocol was not validated separately against optical motion capture in patients with acute complete ACL rupture. Since all patient data were obtained from outpatient clinics during the acute injury phase, participants’ motor capacities were restricted, which limited the analysis to gait assessment only. Subsequent studies could expand the scope to include other rehabilitation evaluations, such as single-leg hop, stair ascent, and descent. The sample size was insufficient to perform subgroup analyses, such as stratification by sex. Future research with larger cohorts should consider including such subgroup analyses to explore potential differences attributable to demographic or injury-related factors.

## Conclusion

Patients with ACL rupture showed gait kinematic alterations involving both lower extremities. The most consistent findings were bilateral reductions in hip external rotation, hip adduction, and ankle internal rotation, together with reduced maximum knee flexion mainly on the affected side. Kinematic deviations on the nonaffected side suggest possible bilateral gait adaptations. These findings may support considering hip and ankle kinematics, in addition to knee kinematics, during preoperative assessment and rehabilitation planning after ACL rupture.

## References

[1] Abdullah M Hulleck AA Katmah R Khalaf K El-Rich M. Multibody dynamics-based musculoskeletal modeling for gait analysis: a systematic review. J Neuroeng Rehabil. 2024;21(1):178. 39369227 10.1186/s12984-024-01458-y PMC11452939

[2] Aghdam HA Haghighat F Rezaie M Kavyani M Karimi MT. Comparison of the knee joint reaction force between individuals with and without acute anterior cruciate ligament rupture during walking. J Orthop Surg Res. 2022;17(1):250. 35505440 10.1186/s13018-022-03136-y PMC9066915

[3] Al-Amri M Nicholas K Button K Sparkes V Sheeran L Davies JL. Inertial measurement units for clinical movement analysis: reliability and concurrent validity. Sensors (Basel). 2018;18(3):719. 29495600 10.3390/s18030719 PMC5876797

[4] Feuvrier F Sijobert B Azevedo C , et al. Inertial measurement unit compared to an optical motion capturing system in post-stroke individuals with foot-drop syndrome. Ann Phys Rehabil Med. 2020;63(3):195-201. 31009801 10.1016/j.rehab.2019.03.007

[5] Filbay SR Grindem H. Evidence-based recommendations for the management of anterior cruciate ligament (ACL) rupture. Best Pract Res Clin Rheumatol. 2019;33(1):33-47. 31431274 10.1016/j.berh.2019.01.018 PMC6723618

[6] Fostiak K Bichowska-Paweska M Trybulski R Alexe DI Wilk M. Acute effects of BFR intra-conditioning on torque and muscle activity of the rectus femoris muscle during isokinetic knee extensions. Sci Rep. 2025;15(1):30312. 40830201 10.1038/s41598-025-15562-z PMC12365154

[7] Fuentes A Hagemeister N Ranger P Heron T de Guise JA. Gait adaptation in chronic anterior cruciate ligament-deficient patients: pivot-shift avoidance gait. Clin Biomech (Bristol, Avon). 2011;26(2):181-187. 10.1016/j.clinbiomech.2010.09.016 20965627

[8] Ghattas J Jarvis DN. Validity of inertial measurement units for tracking human motion: a systematic review. Sports Biomech. 2024;23(11):1853-1866. 34698600 10.1080/14763141.2021.1990383

[9] Hurd WJ Snyder-Mackler L. Knee instability after acute ACL rupture affects movement patterns during the mid-stance phase of gait. J Orthop Res. 2007;25(10):1369-1377. 17557321 10.1002/jor.20440 PMC2859715

[10] Ismail SA Button K Simic M Van Deursen R Pappas E. Three-dimensional kinematic and kinetic gait deviations in individuals with chronic anterior cruciate ligament deficient knee: A systematic review and meta-analysis. Clin Biomech (Bristol, Avon). 2016;35:68-80. 10.1016/j.clinbiomech.2016.04.002 27132248

[11] Kapoor B Clement DJ Kirkley A Maffulli N. Current practice in the management of anterior cruciate ligament injuries in the United Kingdom. Br J Sports Med. 2004;38(5):542-544. 15388535 10.1136/bjsm.2002.002568 PMC1724936

[12] Kaur M Ribeiro DC Theis JC Webster KE Sole G. Movement patterns of the knee during gait following ACL reconstruction: a systematic review and meta-analysis. Sports Med. 2016;46(12):1869-1895. 26936269 10.1007/s40279-016-0510-4

[13] Larwa J Stoy C Chafetz RS Boniello M Franklin C. Stiff landings, core stability, and dynamic knee valgus: a systematic review on documented anterior cruciate ligament ruptures in male and female athletes. Int J Environ Res Public Health. 2021;18(7):3826. 33917488 10.3390/ijerph18073826 PMC8038785

[14] Nouman M Shabnam J Anwar S , et al. Effect of iliotibial band myofascial release combined with valgus correction exercise on pain, range of motion, balance, and quality of life in patients with grade II knee osteoarthritis: a randomized clinical trial. Life (Basel). 2024;14(11):1379. 39598178 10.3390/life14111379 PMC11595761

[15] Petersen W Guenther D Imhoff AB , et al. Management after acute rupture of the anterior cruciate ligament (ACL). Part 1: ACL reconstruction has a protective effect on secondary meniscus and cartilage lesions. Knee Surg Sports Traumatol Arthrosc. 2023;31(5):1665-1674. 35445329 10.1007/s00167-022-06960-1 PMC10089999

[16] Renstrom PA. Eight clinical conundrums relating to anterior cruciate ligament (ACL) injury in sport: recent evidence and a personal reflection. Br J Sports Med. 2013;47(6):367-372. 22942168 10.1136/bjsports-2012-091623

[17] Slater LV Hart JM Kelly AR Kuenze CM. Progressive changes in walking kinematics and kinetics after anterior cruciate ligament injury and reconstruction: a review and meta-analysis. J Athl Train. 2017;52(9):847-860. 28985125 10.4085/1062-6050-52.6.06 PMC5634233

[18] Ursei ME Accadbled F Scandella M , et al. Foot and ankle compensation for anterior cruciate ligament deficiency during gait in children. Orthop Traumatol Surg Res. 2020;106(1):179-183. 31526709 10.1016/j.otsr.2019.07.009

[19] Wang M Lin Z Wang W , et al. Kinematic alterations after anterior cruciate ligament reconstruction via transtibial techniques with medial meniscal repair versus partial medial meniscectomy. Am J Sports Med. 2021;49(12):3293-3301. 34428082 10.1177/03635465211033982

[20] Yona T Peskin B Fischer A. The COMPWALK-ACL: a dataset of multi-pace IMU gait kinematics in adolescents, adults, and ACL injured patients. Sci Data. 2025;13(1):4. 41366255 10.1038/s41597-025-06307-8 PMC12764956

[21] Zeng Z Liu Y Hu X Tang M Wang L. Validity and reliability of inertial measurement units on lower extremity kinematics during running: a systematic review and meta-analysis. Sports Med Open. 2022;8(1):86. 35759130 10.1186/s40798-022-00477-0 PMC9237201

[22] Zhang LQ Shiavi RG Limbird TJ Minorik JM. Six degrees-of-freedom kinematics of ACL deficient knees during locomotion-compensatory mechanism. Gait Posture. 2003;17(1):34-42. 12535724 10.1016/s0966-6362(02)00052-8

[23] Zhang Y Huang W Yao Z , et al. Anterior cruciate ligament injuries alter the kinematics of knees with or without meniscal deficiency. Am J Sports Med. 2016;44(12):3132-3139. 27511793 10.1177/0363546516658026

